# *Notes from the Field:* Unintentional Drug Overdose Deaths with Kratom Detected — 27 States, July 2016–December 2017

**DOI:** 10.15585/mmwr.mm6814a2

**Published:** 2019-04-12

**Authors:** Emily O’Malley Olsen, Julie O’Donnell, Christine L. Mattson, Joshua G. Schier, Nana Wilson

**Affiliations:** 1 Division of Unintentional Injury Prevention, National Center for Injury Prevention and Control, CDC.

Kratom (*Mitragyna speciosa*), a plant native to Southeast Asia, contains the alkaloid mitragynine, which can produce stimulant effects in low doses and some opioid-like effects at higher doses when consumed (*1*). Use of kratom has recently increased in popularity in the United States, where it is usually marketed as a dietary or herbal supplement (*1*). Some studies suggest kratom has potential for dependence and abuse (*1*,*2*). As of April 2019, kratom was not scheduled as a controlled substance. However, since 2012, the Food and Drug Administration has taken a number of actions related to kratom, and in November 2017 issued a public health advisory*; in addition, the Drug Enforcement Administration has identified kratom as a drug of concern. During 2011–2017, the national poison center reporting database documented 1,807 calls concerning reported exposure to kratom (*3*). To assess the impact of kratom, CDC analyzed data from the State Unintentional Drug Overdose Reporting System (SUDORS).

CDC funds 32 states and the District of Columbia to abstract into SUDORS detailed data on unintentional and undetermined intent opioid overdose deaths from death certificates and medical examiner and coroner reports, including postmortem toxicology results.^†^ Although kratom is not an opioid, overdose deaths involving kratom (including nonopioid overdose deaths) are included in SUDORS.^§^ Although postmortem toxicology testing varies in scope among medical examiners and coroners, SUDORS records all substances detected on postmortem toxicology testing, along with overdose-specific circumstances. CDC analyzed overdose deaths in which kratom was detected on postmortem toxicology testing and deaths in which kratom was determined by a medical examiner or coroner to be a cause of death in 11 states during July 2016–June 2017 and in 27 states during July–December 2017.^¶^

Data on 27,338 overdose deaths that occurred during July 2016–December 2017 were entered into SUDORS, and 152 (0.56%) of these decedents tested positive for kratom on postmortem toxicology (kratom-positive). Postmortem toxicology testing protocols were not documented and varied among and within states. Kratom was determined to be a cause of death (i.e., kratom-involved) by a medical examiner or coroner for 91 (59.9%) of the 152 kratom-positive decedents, including seven for whom kratom was the only substance to test positive on postmortem toxicology, although the presence of additional substances cannot be ruled out (*4*).

In approximately 80% of kratom-positive and kratom-involved deaths in this analysis, the decedents had a history of substance misuse, and approximately 90% had no evidence that they were currently receiving medically supervised treatment for pain. Postmortem toxicology testing detected multiple substances for almost all decedents (Table). Fentanyl and fentanyl analogs were the most frequently identified co-occurring substances; any fentanyl was listed as a cause of death for 65.1% of kratom-positive decedents and 56.0% of kratom-involved decedents. Heroin was the second most frequent substance listed as a cause of death (32.9% of kratom-positive decedents), followed by benzodiazepines (22.4%), prescription opioids (19.7%),** and cocaine (18.4%).

**TABLE:** Co-occurrence of substances and circumstances among overdose decedents with kratom detected on postmortem toxicology — State Unintentional Drug Overdose Reporting System, 27 states,* July 2016–December 2017

Characteristic/Circumstance	Kratom detected on toxicology (n = 152) No. (%)	Kratom determined to be a cause of death (n = 91) No. (%)
**Sex**		
Male	116 (76.3)	69 (75.8)
Female	36 (23.7)	22 (24.2)
**Race**		
White^†^	119 (91.5)	81 (93.1)
Nonwhite	11 (8.5)	—^§^
**Medically supervised pain treatment**		
No evidence	138 (90.8)	80 (87.9)
Evidence	14 (9.2)	11 (12.1)
**Previous overdose reported**		
None	139 (91.5)	81 (89.0)
One or more	13 (8.5)	10 (11.0)
**History of substance misuse reported (opioid and/or nonopioid)**		
No evidence	29 (19.1)	20 (22.0)
Evidence	123 (80.9)	71 (78.0)
**Co-occurring substances listed as a cause of death**^¶,^**		
Any fentanyl (including analogs)	99 (65.1)	51 (56.0)
Heroin^††^	50 (32.9)	23 (25.3)
Benzodiazepines	34 (22.4)	24 (26.4)
Prescription opioids^§§^	30 (19.7)	22 (24.2)
Cocaine	28 (18.4)	15 (16.5)
Alcohol	19 (12.5)	11 (12.1)
Methamphetamine	13 (8.6)	—

* Twenty-seven states reported data for the period July 2016–December 2017. Eleven states reported deaths that occurred during the entire period: Kentucky, Maine, Massachusetts, Missouri, New Hampshire, New Mexico, Ohio, Oklahoma, Rhode Island, West Virginia, and Wisconsin. Sixteen additional states only reported deaths that occurred during July–December 2017: Alaska, Connecticut, Delaware, Florida, Georgia, Illinois, Indiana, Minnesota, New Jersey, North Carolina, Pennsylvania, Tennessee, Utah, Vermont, Virginia, and Washington. Data were current as of January 22, 2019.

^†^ Non-Hispanic. Race/ethnicity data were missing for 22 decedents.

^§^ Number of deaths was <10.

^¶^ Identified as a cause of death by a medical examiner or coroner.

** Multiple substances could be listed as a cause of death; therefore, the substances are not mutually exclusive.

^††^ Substances coded as heroin were heroin and 6-monoacetylmorphine. In addition, morphine and codeine were coded as heroin if the scene or other evidence indicated their presence as a result of consumption in conjunction with evidence of heroin use, injection, or illicit drug use, and no evidence of prescribed morphine or codeine.

^§§^ Substances coded as prescription opioids were oxycodone, oxymorphone, hydrocodone, hydromorphone, tramadol, buprenorphine, methadone, meperidine, tapentadol, dextrorphan, levorphanol, propoxyphene, pentazocine, and phenacetin. Also coded as prescription opioids were brand names (e.g., Opana), metabolites (e.g., nortramadol) for these substances, and these substances in combination with nonopioids (e.g., acetaminophen-oxycodone). Morphine and codeine were coded as prescription opioids if the scene or other evidence indicated their presence as a result of consumption of prescription morphine or codeine, rather than as a result of metabolism of or impurities of heroin, respectively. Fentanyl was coded as a prescription opioid if the scene or other evidence indicated likely consumption of prescription fentanyl rather than illicitly manufactured fentanyl. Decedents might have tested positive for other nonopioid substances. This analysis does not distinguish between prescription drugs prescribed to the decedent and those that were diverted.

Kratom-positive deaths accounted for <1% of all SUDORS overdose deaths during July 2016–December 2017. Identification of kratom is method-dependent (*5*); therefore, these data might underestimate the number of kratom-positive deaths, although the extent cannot be determined. However, because SUDORS records results of jurisdiction-specific postmortem toxicology testing, as well as overdose-specific circumstances, it is possible to ascertain that kratom was present primarily in deaths that occurred as a result of overdoses related to substance misuse and that kratom was most often detected in combination with multiple other substances.

The type and number of substances detected in kratom-involved deaths can inform overdose prevention strategies (*6*). Documentation of postmortem toxicology testing protocols is needed to further clarify the extent to which kratom contributes to fatal overdoses.

## Footnotes

* https://www.fda.gov/NewsEvents/PublicHealthFocus/ucm584952.htm.

† Whereas most states in SUDORS submit data on 100% of their unintentional and undetermined intent opioid-involved overdose deaths, Florida, Illinois, Missouri, Pennsylvania, and Washington submit data on a subset of counties that reflect at least 75% of drug overdose deaths in the state.

§ SUDORS records data on fatal unintentional and undetermined intent overdoses in which at least one opioid contributed to death, as well as fatal overdoses with no contributing opioid, if substances that have opioid-like properties (currently, kratom is the only such substance) contributed to death. For all included deaths, SUDORS records all substances testing positive on postmortem toxicology testing (including those that did and did not contribute to death).

¶ Twenty-seven states reported data for the period July 2016–December 2017. Eleven states reported deaths that occurred during the entire period: Kentucky, Maine, Massachusetts, Missouri, New Hampshire, New Mexico, Ohio, Oklahoma, Rhode Island, West Virginia, and Wisconsin. Sixteen additional states only reported deaths that occurred during July–December 2017: Alaska, Connecticut, Delaware, Florida, Georgia, Illinois, Indiana, Minnesota, New Jersey, North Carolina, Pennsylvania, Tennessee, Utah, Vermont, Virginia, and Washington. Data were current as of January 22, 2019.

** Substances coded as prescription opioids were oxycodone, oxymorphone, hydrocodone, hydromorphone, tramadol, buprenorphine, methadone, meperidine, tapentadol, dextrorphan, levorphanol, propoxyphene, pentazocine, and phenacetin. Also coded as prescription opioids were brand names (e.g., Opana), metabolites (e.g., nortramadol) of these substances, and these substances in combination with nonopioids (e.g., acetaminophen-oxycodone). Morphine and codeine were coded as prescription opioids if the scene or other evidence indicated their presence as a result of consumption of prescription morphine or codeine, rather than as a result of metabolism of or impurities of heroin, respectively. Fentanyl was coded as a prescription opioid if the scene or other evidence indicated likely consumption of prescription fentanyl rather than illicitly manufactured fentanyl. Decedents might have tested positive for other nonopioid substances. This analysis does not distinguish between prescription drugs prescribed to the decedent and those that were diverted.
